# Perception of Arabidopsis *At*Pep peptides, but not bacterial elicitors, accelerates starvation-induced senescence

**DOI:** 10.3389/fpls.2015.00014

**Published:** 2015-01-23

**Authors:** Kay Gully, Tim Hander, Thomas Boller, Sebastian Bartels

**Affiliations:** Department of Environmental Sciences, Botany, Zürich-Basel Plant Science Center, University of BaselBasel, Switzerland

**Keywords:** Plant elicitor peptide (Pep), starvation, senescence, MAMP, Arabidopsis, PEPR, PTI

## Abstract

Members of the *At*Pep group of Arabidopsis endogenous peptides have frequently been reported to induce pattern-triggered immunity (PTI) and to increase resistance to diverse pathogens by amplifying the innate immune response. Here, we made the surprising observation that dark-induced leaf senescence was accelerated by the presence of Peps. Adult leaves as well as leaf discs of Col-0 wild type plants showed a Pep-triggered early onset of chlorophyll breakdown and leaf yellowing whereas *pepr1 pepr2* double mutant plants were insensitive. In addition, this response was dependent on ethylene signaling and inhibited by the addition of cytokinins. Notably, addition of the bacterial elicitors flg22 or elf18, both potent inducers of PTI, did not provoke an early onset of leaf senescence. Continuous darkness leads to energy deprivation and starvation and therewith promotes leaf senescence. We found that continuous darkness also strongly induced *PROPEP3* transcription. Moreover, Pep-perception led to a rapid induction of *PAO*, *APG7*, and *APG8a*, genes indispensable for chlorophyll degradation as well as autophagy, respectively, and all three hallmarks of starvation and senescence. Notably, addition of sucrose as a source of energy inhibited the Pep-triggered early onset of senescence. In conclusion, we report that Pep-perception accelerates dark/starvation-induced senescence via an early induction of chlorophyll degradation and autophagy. This represents a novel and unique characteristic of PEPR signaling, unrelated to PTI.

## Introduction

Plant elicitor peptides (Peps) have been identified as endogenous elicitors of the plant's immune system (Huffaker et al., 2006). In Arabidopsis perception of Peps by their two receptors, PEP-RECEPTOR1 (PEPR1) and PEPR2, triggers a set of immune responses known as pattern-triggered immunity (PTI) (Krol et al., 2010; Yamaguchi et al., 2010; Bartels et al., 2013). Due to their immunity-inducing activity and their endogenous origin they have been classified as damage- or danger-associated molecular patterns (DAMPs), analogous to the exogenous elicitors of microbial origin, the microbe-associated molecular patterns (MAMPs) (Boller and Felix, 2009). Multiple studies connected Peps with the induction and amplification of plant immunity against very diverse pathogens including bacteria, fungi, and herbivores (Huffaker et al., 2006, 2013; Yamaguchi et al., 2010; Liu et al., 2013; Tintor et al., 2013).

Peps originate from the assumed cleavage of small precursor proteins called PROPEPs (Huffaker et al., 2006). They are encoded in the C-terminal end of these PROPEPs and comprise between 23 and 29 amino acids (Huffaker and Ryan, 2007; Bartels et al., 2013). Notably, in contrast to systemins, classic elicitor peptides from solanaceous species, they have been identified in several plant species including dicots (e.g., Arabidopsis, potato and soy bean) and monocots (maize, rice, and sorghum) (Ryan and Pearce, 2003; Huffaker et al., 2013).

In Arabidopsis eight *PROPEP* genes are encoded in the genome. Their promoters show distinct activity patterns ranging from a strict limitation to the root tips (*PROPEP4* and *PROPEP7*) to a rather widespread activation in roots and leaves (*PROPEP5*) (Bartels et al., 2013). A comprehensive co-expression analysis indicated that only the transcription of *PROPEP1*, *PROPEP2*, and *PROPEP3* is significantly co-regulated with the one of defense-related genes whereas other *PROPEPs* showed strongest transcriptional induction upon abiotic stress as well as developmental programs (Bartels et al., 2013). However, perception of all eight peptides elicits similar PTI-like responses, indicating a redundant function in activating PEPR-mediated signaling (Huffaker and Ryan, 2007; Bartels et al., 2013).

The final phase of plant development is called senescence. In annual plants like Arabidopsis senescence is seen as a program to relocalize nutrients from leaves into seeds and often starts with the transition of the vegetative to the reproductive phase (Thomas, 2013; Avila-Ospina et al., 2014). Leaf senescence is therefore controlled by developmental signals mediated by plant hormones. Especially cytokinins and ethylene have been linked to senescence with antagonistic roles. Treatment of plants with ethylene induces ripening of fruits and the rapid onset of leaf senescence (Graham et al., 2012), whereas mutants impaired in ethylene signaling exhibit a delayed senescence phenotype (Li et al., 2013). In contrast, cytokinins are thought to be the “foliar fountain of youth” since the application of cytokinins blocks senescence and promotes longevity (Zwack and Rashotte, 2013). Consistently, cytokinin levels were shown to be high in young leaves and continuously decrease during developmental senescence (Lim et al., 2007).

Beside internal factors also external factors promote or delay the onset of leaf senescence. One of these external factors is the availability of nutrients. Abundance of for example nitrogen is known to delay senescence whereas periods of starvation can lead to premature leaf senescence (Masclaux-Daubresse et al., 2010; Balazadeh et al., 2014). Starvation is based either on a shortage of essential elements like nitrogen, sulfur or phosphorus or the lack of energy due to shading or prolonged darkness (Lim et al., 2007; Baena-Gonzalez and Sheen, 2008; Avila-Ospina et al., 2014). During starvation-induced senescence, a nutrient remobilization program is started to maintain important cellular functions. In source leaves, this includes an induction of autophagy as well as the degradation of chloroplast proteins and chlorophyll, the latter a visible sign of senescence; if the situation of starvation persists, the leaves finally die (Lim et al., 2007; Liu and Bassham, 2012). Continuous darkness is one way to create a situation of starvation and induce leaf senescence. It has been especially investigated in detached or individually darkened leaves and is commonly known as dark-induced senescence (Weaver and Amasino, 2001; Zhou et al., 2011).

In our present study we report the acceleration of dark-induced senescence due to the perception of AtPeps. Remarkably, we found that senescence acceleration is a specific response to Pep-perception and not a pleiotropic effect of PTI activation. We further investigated the mechanism behind the senescence phenotype and found that Pep-perception triggered a rapid induction of genes encoding key factors of chlorophyll breakdown and autophagy. Thus, we conclude that the Pep-PEPR system might fine-tune the nutrient remobilization response activated upon continuous darkness and subsequent energy depletion and starvation.

## Materials and methods

### Plant material

The Arabidopsis (*Arabidopsis thaliana*) plants used in this study were grown on soil as one plant per pot at 21°C and an 8-h photoperiod for 4–5 weeks. All mutants used in this study are based on the Columbia (Col-0) ecotype. The *pepr1 pepr2* double mutant has been described by Flury et al. (2013), the *ein3 eil1* double mutant by Alonso et al. (2003).

### Peptides

The peptides flg22 (QRLSTGSRINSAKDDAAGLQIA), AtPep1 (ATKVKAKQRGKEKVSSGRPGQHN), AtPep2 (DNKAKSKKRDKEKPSSGRPGQTNSVPNAAIQVYKED), AtPep3 (EIKARGKNKTKPTPSSGKGGKHN), and elf18 (Ac-SKEKFERTKPHVNVGTIG), obtained from EZBiolabs, were dissolved in a solution containing 1 mg mL^−1^ bovine serum albumin and 0.1 M NaCl. These 100 μM peptide stock solutions were further diluted in water or the respective assay solution to reach the final concentration.

### Dark-induced senescence assay with and without supplementation

Assays with whole leaves: All leaves were detached and floated individually on distilled, deionized water in separate petri dishes. All dishes were sealed and wrapped in aluminum foil and kept for 4 days in the growth chamber.

Assays with leaf discs: Leaf discs (1 cm diameter) were punched out from the third and fourth true rosette leaves of individual plants. Discs (4 replicates) were floated in petri dishes on distilled, deionized water supplied as indicated with elicitor peptides, 0.5% sucrose, 50 nM trans-Zeatin or respective control solutions. Petri dishes were sealed with Parafilm tape, wrapped with aluminum foil and placed back into the growth chamber for the indicated number of days.

### Chlorophyll extraction

Total chlorophyll concentration of single leaf discs were determined in 80% acetone extracts and calculated using equations and specific extinction coefficients as reported by Lichtenthaler (1987).

### Quantitative reverse transcription-PCR

For each sample, total RNA of seven Arabidopsis leaf discs was extracted with the NucleoSpin RNA plant extraction kit (Macherey-Nagel), using recombinant DNase according to the manufacturer's specification. Per PCR reaction, complementary DNA was synthesized from 10 ng of RNA with oligo(dT) primers using the AMV reverse transcription kit (Promega) according to the manufacturer's instructions. Quantitative RT-PCR was performed in a 96-well format using a LightCycler® 480 Instrument (Roche). On the basis of the obtained threshold cycle values, normalized expression to the reference gene *UBIQUITIN10* (UBQ10, AT4G05320) was calculated using the qGene protocol (Muller et al., 2002). The gene-specific primers used were as follows: *UBQ10* (AT4G05320) with UBQ_fw (5′ GGCCTTGTATAATCCCTGATGAATAAG) and UBQ_rv (5′ AAAGAGATAACAGGAACGGAAACATAG), *APG7* (AT5G45900) with APG7_fw (5′ ATTGGCGCGACTCAGATTTAAC), and APG7_rv (5′ CCATCTTGAGCGAATCAGTGC), *APG8A* (AT4G21980) with APG8A_fw (5′ CTTGAAATTCGCAGAGACTAATCG), and APG8A_rv (5′ ACTTTGTCCAGCCTTCTCCAC), *PAO* (AT3G44880) with PAO_fw (5′ CTAAATCAATGGAGTCACCAGAC), and PAO_rv (5′ TCTATCTAGCATCTGACGCTTG), *PROPEP1* (AT5G64900) with PP1_qRT_fw (5′-ATCAGATAGACGAAGCGAAG), and PP1_qRT_rv (5′-CTAATTATGTTGGCCAGGAC), *Propep3* (At5G64905) with PP3_qRT_fw (5′-CAACGATGGAGAATCTCAGA), and PP3_qRT_rv (5′-CTAATTGTGTTTGCCTCCTTT).

### Statistical analyses

Statistical significance was calculated using the online available “GraphPad Quickcals *t*-test calculator” tool (http://www.graphpad.com/quickcalcs/ttest1/?Format=SEM). Nummerical values used for the calculations of an unpaired *t*-test are provided in Data Sheet 1 in Supplementary Material.

## Results

### Perception of *At*Peps accelerates dark-induced senescence

**Figure 1:**
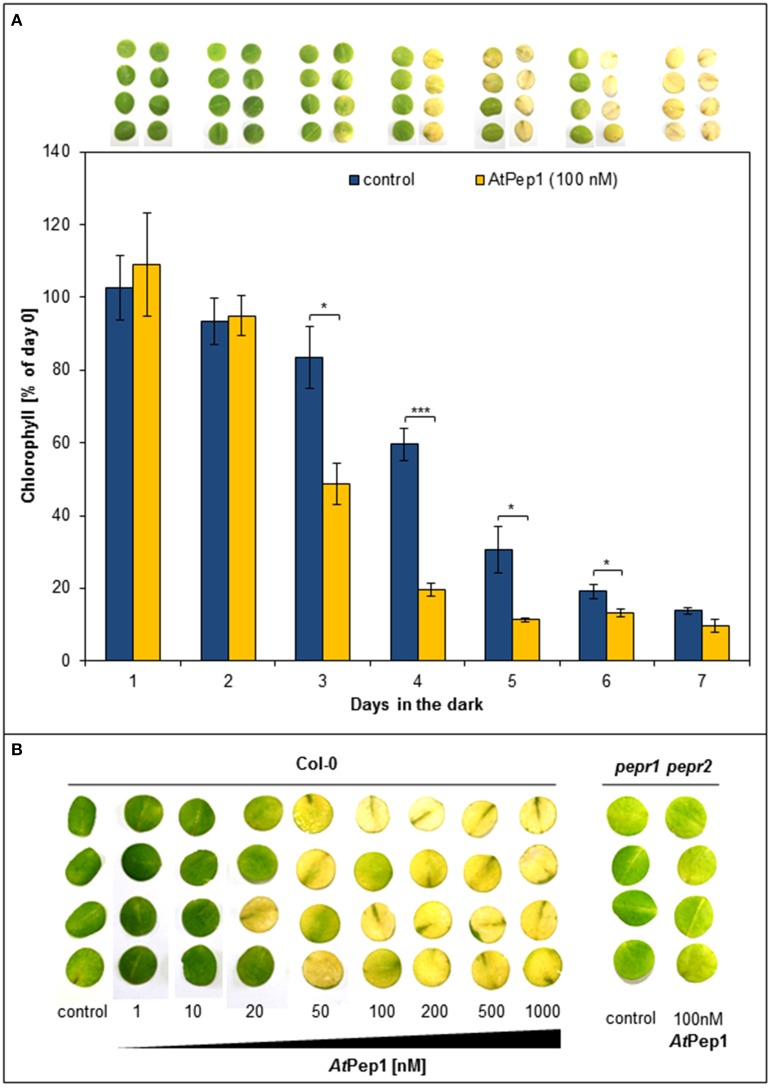
**Perception of Peps accelerates dark-induced senescence in a concentration and PEPR-dependent manner. (A)** Four leaf discs of Col-0 plants were floated on water supplemented with 100 nM AtPep1 or without any peptide (control) and kept in darkness for the indicated time. Pictures show the discs after the incubation and bars represent the mean values of extracted chlorophyll of the four discs relative to the mean of untreated “day 0” discs. Error bars show ±1 SE of the mean and asterisks represent Student's *t*-test results (^*^ = *p* < 0.05, ^***^ = *p* < 0.001). **(B)** Leaf discs of Col-0 and *pepr1 pepr2* were treated with the indicated concentrations of AtPep1 or without any peptide (control) and kept for 4 days in darkness before taking the pictures.

In earlier work, we used leaf discs to measure PTI responses like ROS (reactive oxygen species), ethylene production, or MAPK activation; in case of ROS analyses, the leaf discs were kept one night in darkness beforehand, since ROS production upon elicitor perception is blocked in freshly cut discs (Flury et al., 2013). In the context of such ROS experiments, we observed, that wildtype Col-0 discs treated with AtPep1 turned yellow more quickly than untreated discs when kept in continuous darkness. Thus, we started to carefully characterize this surprising effect. As shown in Figure 1A, discs treated with 100 nM AtPep1 showed a first strong reduction in chlorophyll content already after 3 days of continuous darkness and turned yellow after 4 days. In contrast, the chlorophyll content of untreated discs dropped significantly only after 4 days and all discs turned yellow only after 7 days of continuous darkness (Figure 1A). We then tested the dose-response relationship of this effect and found a clear promotion of yellowing already at 20 nM of AtPep1 and saturation between 50 and 100 nM AtPep1 (Figure 1B). These concentrations reflect the ones used to show the effectiveness of AtPep1 and other plant Peps to increase plant resistance to pathogens (Yamaguchi et al., 2010; Huffaker et al., 2013). Notably, the acceleration of dark-induced leaf yellowing upon Pep treatment was fully dependent on functional Pep-signaling since the *pepr1 pepr2* double mutant did not respond to the presence of AtPep1 but displayed a yellowing pattern comparable to the Col-0 discs (Figure 1B). Thus, PEPR signaling leads to the acceleration of dark-induced senescence.

### Acceleration of senescence by Peps depends on leaf age

**Figure 2:**
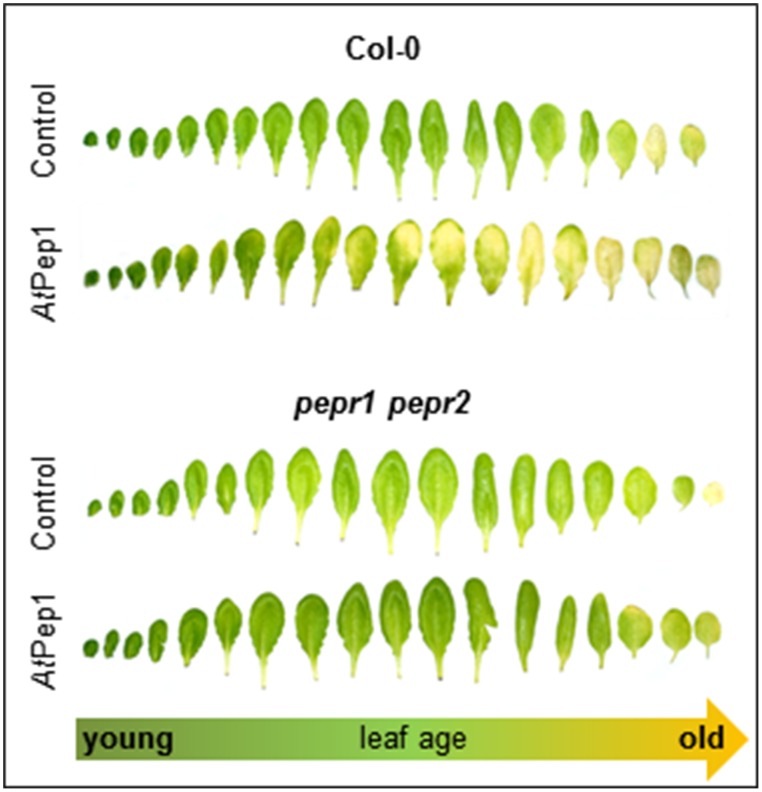
**PEPR-mediated acceleration of senescence depends on leaf age**. Pictures show all leaves of representative Col-0 and *pepr1 pepr2* plants, after 4 days incubation in darkness floating on a solution with 100 nM AtPep1 or without any peptide (control).

Next we used whole detached leaves instead of leaf discs to see if whole leaves show a similar behavior. Moreover, since we generally use only fully expanded mature leaves for leaf disc preparation we were also interested if leaf age has an impact on the Pep-triggered acceleration of dark-induced senescence. Thus, we harvested all leaves from single plants and floated them on water with or without AtPep1 supplementation. Each leaf was sealed in a separate petri dish to exclude an influence of volatiles like ethylene, which is produced upon AtPep perception (Bartels et al., 2013; Flury et al., 2013). As shown in Figure 2, most of the adult leaves showed signs of senescence when incubated for 4 days in darkness floating on a 100 nM AtPep1 solution. In contrast, floating the leaves in water without addition of AtPep1 produced only signs of senescence in the oldest three leaves (Figure 2). The youngest, still expanding leaves were not affected by Pep-treatment.

### Ethylene and cytokinins act antagonistically on the Pep-triggered acceleration of senescence

Age-dependent senescence is enhanced by ethylene signaling whereas it is suppressed by addition of cytokinins (Li et al., 2013; Zwack and Rashotte, 2013). Thus, we investigated the impact of these senescence-related plant hormones on the Pep-accelerated senescence. Here, we switched back to the use of leaf discs since we noted that the discs responded more equally to the addition of AtPep1 than whole leaves, probably due to the better accessibility of the disc tissue. In whole leaves Peps seem to enter mainly through the cut petiole which tends to be quickly blocked by vascular embolisms (Huffaker et al., 2006).

**Figure 3:**
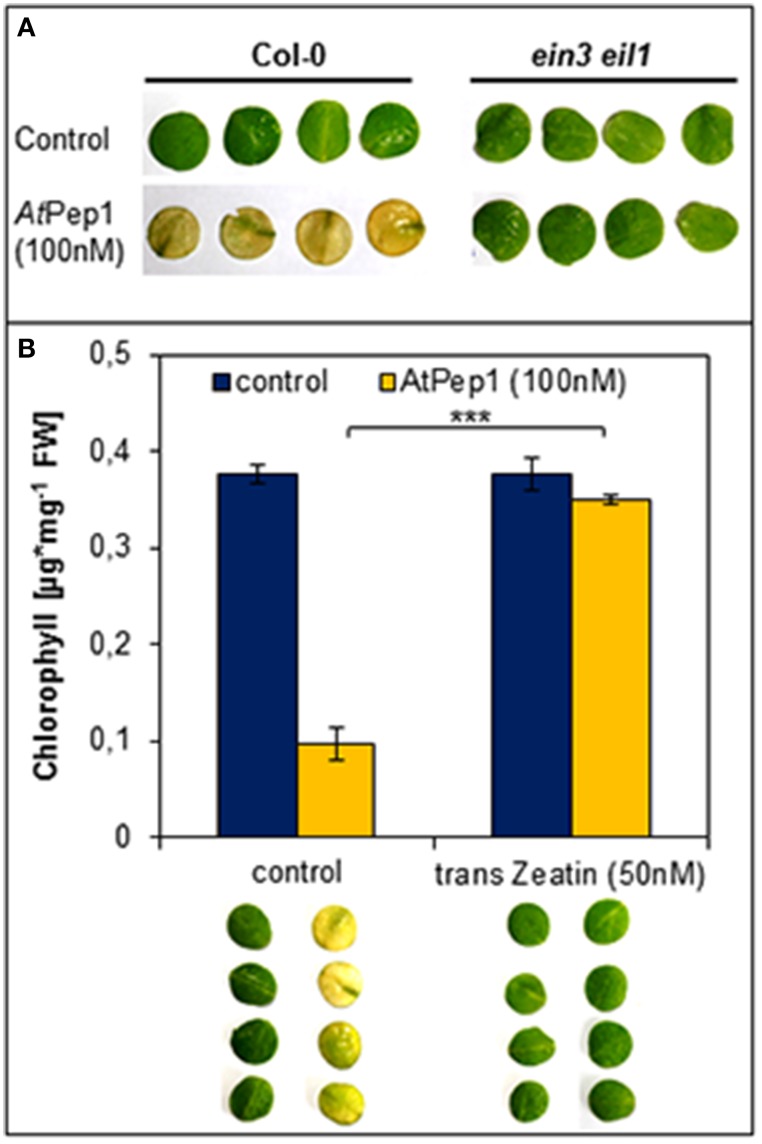
**Antagonistic action of ethylene and cytokinins on the Pep-triggered acceleration of dark-induced senescence. (A)** Leaf discs of Col-0 as well as *ein3 eil1* were incubated for 4 days in darkness floating on a solution with 100 nM AtPep1or without any peptide (control). **(B)** Leaf discs of Col-0 plants were treated with AtPep1 (100 nM) solution or without any peptide (control), and a second set of discs was co-treated with 50 nM trans-Zeatin. Pictures show the discs after 4 days incubation in darkness. Bars represent the mean of total chlorophyll extracted from four leaf discs. Error bars indicate ±1 SE of the mean and asterisks represent Student's *t*-test results (^***^ = *p* < 0.001).

We used the *ein3 eil1* double mutant, which is fully blocked in ethylene signaling, to investigate the impact of ethylene signaling on the early onset of dark-induced senescence in Pep-treated leaf discs. As shown in Figure 3A, we found that the Pep-treated leaf discs of *ein3 eil1* remained green unlike the Col-0 treated tissue which turned yellow. Thus, functional ethylene signaling appears to be indispensable for an early onset of dark-induced senescence upon Pep-perception.

Regarding cytokinins, addition of as little as 50 nM trans-zeatin is sufficient to suppress the early onset of dark-induced senescence triggered by Pep-perception (Figure 3B). Thus, despite the presence of AtPep1, dark-induced senescence is delayed after activation of cytokinin-signaling. Taken together the early onset of dark-induced senescence upon Pep-perception depends on the status of the senescence-regulating plant hormones ethylene and cytokinins. It is possible that young leaves do not show the Pep-triggered acceleration of dark-induced senescence (see Figure 2) due to higher internal levels of cytokinins (Zwack and Rashotte, 2013).

### Addition of MAMPs does not lead to an early onset of dark-induced senescence

**Figure 4:**
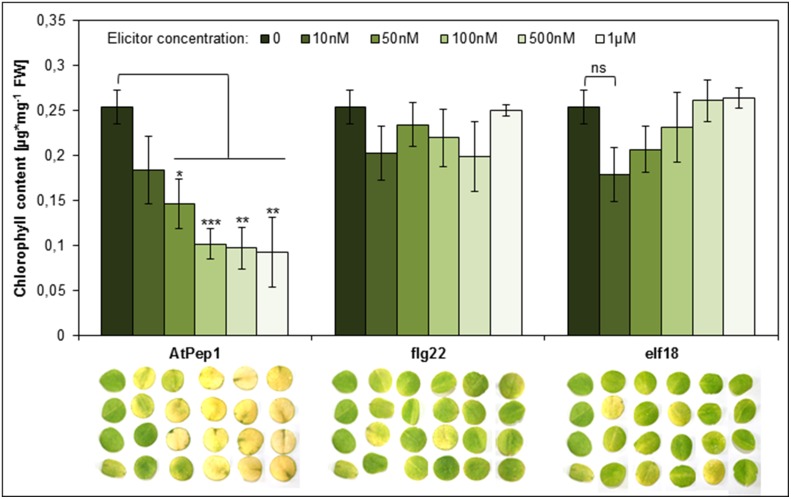
**Perception of AtPep1 but not of the MAMPs flg22 or elf18 accelerates dark-induced senescence**. Four leaf discs of Col-0 plants were floated on water supplemented with the indicated elicitor peptides or without any peptide (0) and kept for 4 days in continuous darkness. Bars show mean values of the total chlorophyll content of four leaf discs. Error bars represent ±1 SE of the mean and asterisks display Student's *t*-test results (^*^ = *p* > 0.05, ^**^ = *p* < 0.01, ^**^ = *p* < 0.001, ns = not significant).

Perception of Peps triggers a PTI-like spectrum of responses similar to the ones elicited by the recognition of MAMPs like flg22 or elf18 (Boller and Felix, 2009; Bartels et al., 2013). Notably, Peps and MAMPs both activate the release of ethylene and functional ethylene signaling is necessary for full activation of defense responses triggered by Peps and MAMPs (Liu et al., 2013; Tintor et al., 2013). However, MAMPs are significantly more potent in inducing PTI including the production and release of ethylene (Flury et al., 2013). Thus, we reasoned that the acceleration of dark-induced senescence might be a pleiotropic effect of activated PTI and ethylene production and thus might also be visible or even more pronounced after addition of the bacterial elicitors flg22 or elf18, which are known to induce a rather strong PTI (Boller and Felix, 2009; Flury et al., 2013). To our surprise neither the use of flg22 nor elf18 led to visible yellowing of the leaf discs or a significant reduction of chlorophyll content (Figure 4). Even at concentrations as high as 1 μM for flg22 or elf18, leaf discs remained as green as controls (Figure 4). In the same experiment concentrations of AtPep1 as little as 50 nM induced the early onset of senescence (Figure 4). Thus, it seems that the acceleration of dark-induced senescence is a specific response upon activation of PEPR signaling and not a pleiotropic effect of PTI activation. This is a surprising and unique feature of the Pep-PEPR signaling system.

### Transcript levels of *PROPEP3* rise during continuous darkness

**Figure 5:**
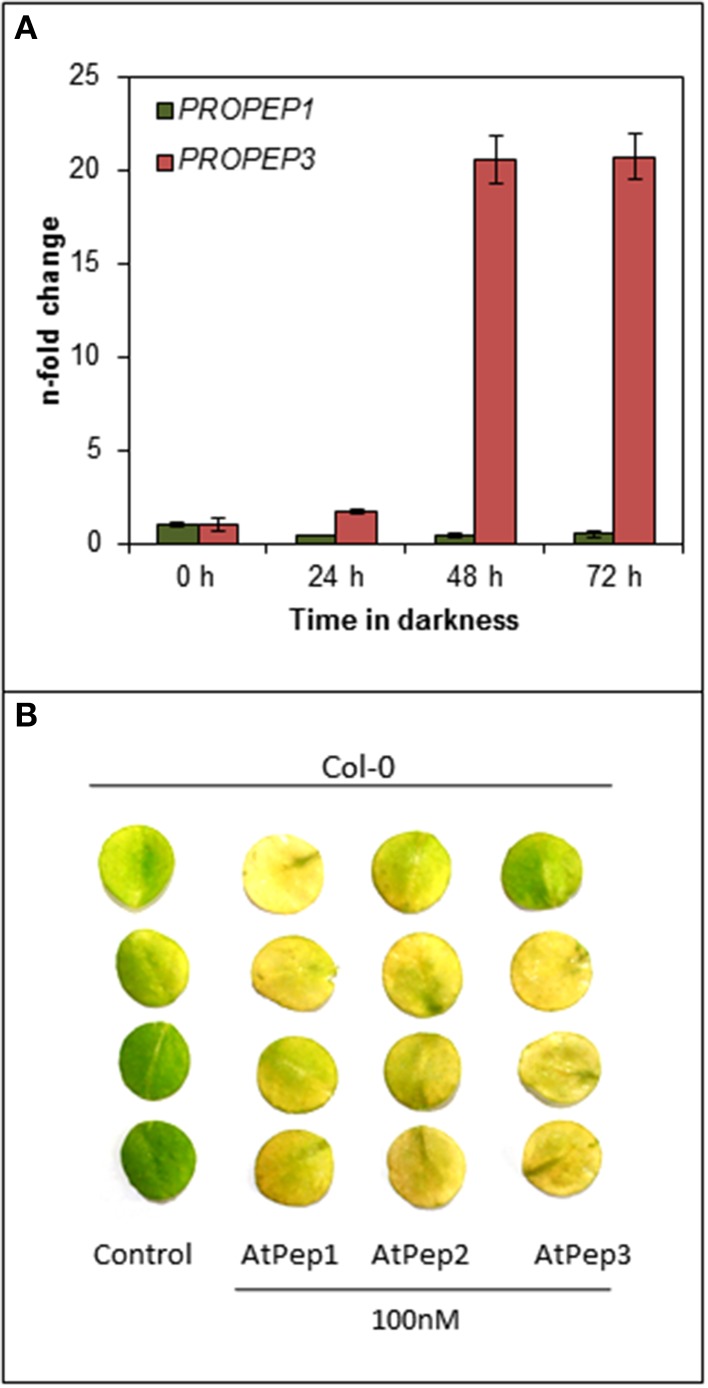
***PROPEP3* transcription is induced during continuous darkness. (A)** Col-0 leaf discs were incubated for the indicated time in darkness floating on water. *PROPEP1* and *PROPEP3* transcript levels were determined by normalizing to *UBQ10* transcripts, and bars indicate the fold change of transcript levels relative to time 0 h. Error bars show the relative ±1 SE of the mean. **(B)** Four leaf discs of Col-0 plants were floated on water supplemented with the indicated elicitor peptides or without any peptide (0) and kept for 4 days in continuous darkness before taking the pictures.

After realizing that the early onset of senescence is a Pep-specific effect we wondered about its biological significance. Since we did not observe any senescence-related phenotype of the *pepr1 pepr2* double mutant during this study (Figures 1, 2), we assumed that this effect might be limited to certain circumstances where Peps are indeed released. Currently these circumstances are unknown but several reports work with the assumption that a loss of cellular integrity during mechanical damage or damage produced by the invasion of pathogens promotes a release of Peps into the apoplast (Huffaker and Ryan, 2007; Boller and Felix, 2009; Yamaguchi and Huffaker, 2011; Bartels et al., 2013; Ross et al., 2014). Since most of the PROPEPs, the precursors of Peps, are only little expressed in leaves but induced upon a certain trigger like wounding or perception of pathogens, we investigated the transcriptional response of *PROPEP1* and *PROPEP3* in leaf discs kept in continuous darkness. The experimental setup was similar to the one used to determine dark-induced senescence and we followed *PROPEP* transcription for 72 h. As shown in Figure 5A *PROPEP1* remained at baseline levels during continuous darkness whereas *PROPEP3* was significantly induced. Transcript levels of *PROPEP3* rose already 2-fold after 24 h of darkness and reached a 20-fold increase after 48 h of darkness. This indicates a connection between the Pep3-PEPR system and stress signaling upon continuous darkness.

It has been shown before, by work of others and ourselves, that Peps redundantly trigger PEPR-mediated signaling, especially PEPR1 recognizes all eight AtPeps and the perception of each peptide results in a similar response (Huffaker and Ryan, 2007; Bartels et al., 2013). Since *PROPEP3* and not *PROPEP1* transcription is strongly induced during continuous darkness we determined if AtPep3 perception triggers the same acceleration of dark-induced senescence as AtPep1 perception did. Indeed, treatment with either AtPep2 or AtPep3 led to the early onset of leaf yellowing comparable to the response upon AtPep1 treatment (Figure 5B). In conclusion, during continuous darkness at least *PROPEP3* transcript levels rose, providing first evidence that the Pep-PEPR system is involved in the response to continuous darkness. Like AtPep1, AtPep3 is capable to induce the early onset of senescence via PEPR1 signaling.

### The Pep-triggered early onset of dark-induced senescence depends on leaf energy status

**Figure 6:**
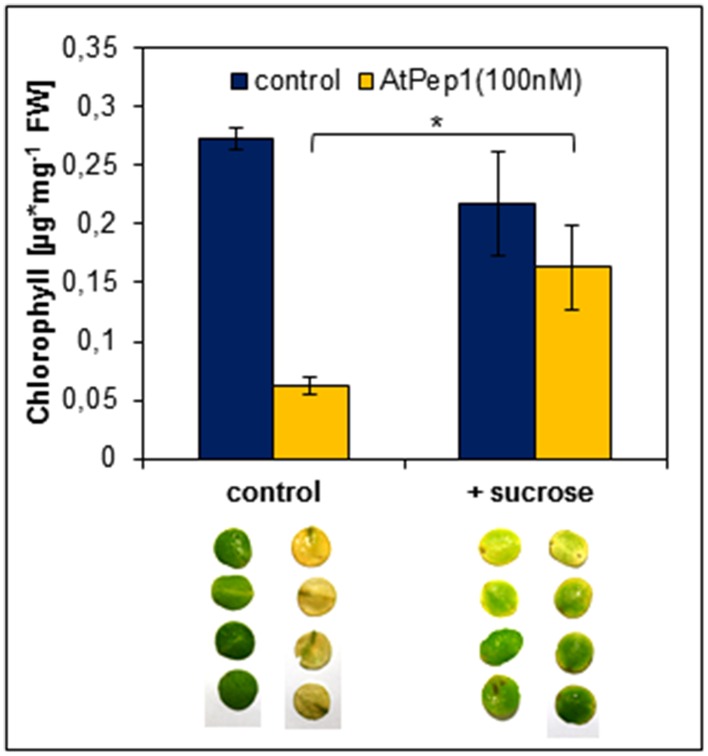
**Sucrose supplementation inhibits the Pep-triggered acceleration of senescence**. Leaf discs of Col-0 plants were floated on a 100 nM AtPep1 solution or on a solution without any peptide (control). Solutions were supplemented with 0.5% sucrose. Pictures show leaf discs after 4 days incubation in darkness. Bars show mean values of the total chlorophyll content of four leaf discs. Error bars indicate ±1 SE of the mean and asterisks represent Student's *t*-test results (^*^ = *p* < 0.05).

Dark-induced senescence is based on energy deprivation that triggers a starvation response, activates autophagy and eventually leads to senescence (Weaver and Amasino, 2001; Rose et al., 2006; Baena-Gonzalez and Sheen, 2008; Liu and Bassham, 2012). Based on our results simultaneous perception of Peps seems to accelerate the chronology of these events. Thus, we tested if a supplementation of energy in form of sucrose could delay the early onset of leaf yellowing upon Pep-perception. It has been reported before that the addition of sucrose to dark-incubated leaves inhibits the induction of genes connected to leaf senescence (Fujiki et al., 2001). Therefore, we performed the same assay as before but supplemented the assay solution with additional 0.5% sucrose. As shown in Figure 6, chlorophyll degradation and leaf yellowing was significantly inhibited by the addition of sucrose despite the simultaneous addition of *At*Pep1. We conclude that the energy status of the tissue has a major impact on the acceleration of dark-induced senescence upon Pep-perception.

### Pep-perception triggers a rapid induction of genes involved in chlorophyll breakdown and autophagy

We had a closer look at the events induced by the advent of starvation and the impact of Pep-perception on these events.

**Figure 7:**
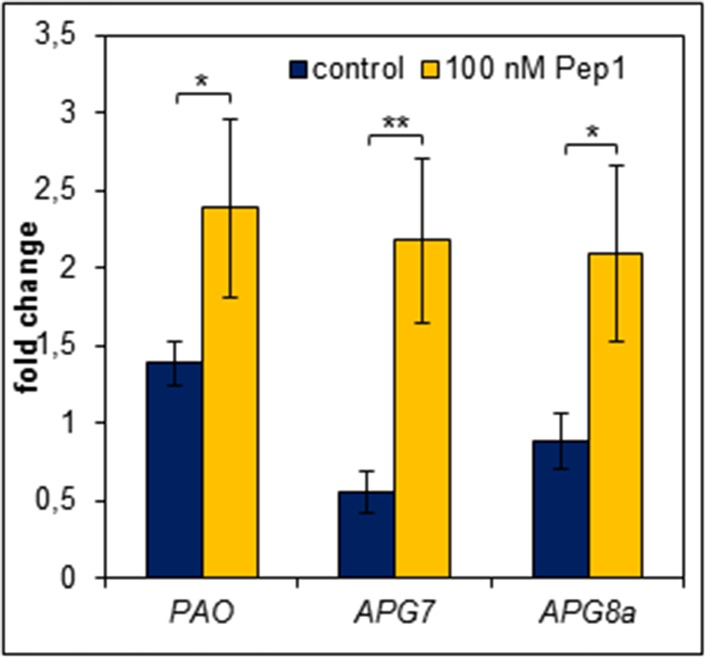
**Pep perception leads to an early induction of genes required for chlorophyll degradation and autophagy**. Leaf discs were treated with 100 nM AtPep1 or without any peptide and incubated in darkness for 24 h prior to harvest. Transcript levels were determined by normalizing to *UBQ10* and calculating the mean of five independent biological replicates. Bars indicate the fold change of transcript levels related to an untreated 0 h control sample. Error bars show the relative ±1 SE of the mean and asterisks represent Student's *t*-test results (^*^ = *p* < 0.05, ^**^ = *p* < 0.01).

In order to survive a situation of starvation plants start to remobilize nutrients by degradation of expendable compounds, structures and organelles (Guiboileau et al., 2012; Liu and Bassham, 2012). Chloroplasts are main sources of carbon (starch) and nitrogen (proteins) which are remobilized during senescence. Their decay is regarded as a first sign of beginning developmental as well as starvation-induced senescence (Diaz et al., 2008; Avila-Ospina et al., 2014). Linked to chloroplast decay is the degradation of chlorophyll which is catalyzed by several enzymes including pheide a oxygenase (PaO) (Hörtensteiner and Kräutler, 2011). Notably, PaO activity is only found during senescence (Pruzinska et al., 2003). Thus, we investigated the transcriptional regulation of *PAO* upon Pep-perception as an early marker of the onset of senescence and nutrient remobilization. As shown in Figure 7 *PAO* transcript levels rose only slightly (1.4 fold, relative to 0 h time) in the untreated leaf discs after 24 h of darkness. In contrast, simultaneous supplementation with AtPep1 induced a significant 2.5 fold rise in *PAO* transcript levels (Figure 7). Thus, activation of PEPR signaling promotes an accelerated chlorophyll breakdown most likely through the stronger induction of *PaO*, encoding the key enzyme for chlorophyll degradation.

A further aspect of the starvation response is autophagy, which appears to be central to nutrient availability during starvation and nutrient remobilization during senescence (Rose et al., 2006; Avila-Ospina et al., 2014; Ren et al., 2014). It has been reported to contribute to starch as well as bulk protein degradation (Thompson and Vierstra, 2005; Wang et al., 2013). Vice versa *APG* genes (their products are building the autophagy machinery) are regulated by carbon and nitrogen status (Rose et al., 2006). Moreover, mutants impaired in proper autophagy were frequently reported to show accelerated developmental as well as starvation-induced senescence (Doelling et al., 2002; Hanaoka et al., 2002; Thompson et al., 2005; Li et al., 2014). Thus, we also measured transcript levels of genes central to autophagy (*APG7* and *APG8a*). Notably both have been shown before to be induced by continuous darkness (Avila-Ospina et al., 2014). Indeed, similar to *PAO* both genes were induced more than 2-fold after 24 h of darkness in the presence of AtPep1 whereas in the absence of AtPep1 we could not detect any induction of *APG7* and *APG8a* (Figure 7).

Finally we also looked into publicly available microarray data to investigate if perception of AtPeps induces more genes related to senescence (Ross et al., 2014). Indeed several genes linked to senescence including WRKY transcriptions factors as well as SAGs are upregulated within 2 h after treatment with AtPep1 (Data Sheet 2 in Supplementary Material) supporting a potential role of PEPR signaling in the onset of senescence.

Taken together, during continuous darkness, Pep-perception triggers an earlier and/or stronger induction of genes encoding enzymes central for chlorophyll breakdown and autophagy. This is most likely the driving force behind the observed Pep-triggered acceleration of dark-induced senescence.

## Discussion

### Acceleration of starvation-induced senescence is a unique feature of PEPR signaling

It is well-established that the Pep-PEPR system is necessary for full immunity against diverse pathogens including bacteria, fungi as well as herbivores (Huffaker et al., 2006, 2013; Yamaguchi et al., 2010; Liu et al., 2013; Tintor et al., 2013). In addition it has recently also been connected to systemic immunity (Ross et al., 2014). However, there are several hints that made us wonder already for many years if this system might have additional functions beside the amplification of plant immunity. First, phylogenetic analysis showed that unlike the receptors for the bacterial MAMPs flg22 or elf18, FLS2 and EFR, respectively, both PEPRs cluster together in the leucine-rich repeat receptor-like kinase subfamily XI with receptors involved in plant development and differentiation (Yamaguchi et al., 2010). Thus, they likely evolved from receptors regulating plant development and might still operate signaling pathways involved in plant development in addition to the PTI-inducing pathways. Second, only some of the *PROPEP* promoters are responsive to biotic stress whereas others appear insensitive to this type of stress (Huffaker et al., 2006; Bartels et al., 2013). Third, a comprehensive co-expression analysis of *PROPEP* transcription upon diverse stresses indicated that only some are co-regulated with genes linked to defense, whereas others showed e.g., regulatory patterns similar to genes involved in reproduction (Bartels et al., 2013).

Leaf senescence is a response that is influenced by multiple external as well as internal factors and thereby integrates stress and developmental signaling (Lim et al., 2007). After our observation that the presence of AtPeps accelerates dark-induced senescence we first thought that this is a pleiotropic effect of the induction of PTI. Immunity is a costly response which could easily aggravate a situation of starvation e.g., during continuous darkness and therewith promote senescence (Buchanan-Wollaston et al., 2005; Baena-Gonzalez and Sheen, 2008; Denance et al., 2013). This goes in line with the effect of sucrose supplementation as a source of energy that blocked the early onset of leaf senescence in the Pep-treated samples. Moreover, activation of PTI includes the release of ethylene, which is known to induce senescence (Bleecker and Kende, 2000; Boller and Felix, 2009). To our surprise addition of strong elicitors of PTI, the MAMPs flg22 and elf18, did not lead to an acceleration of dark-induced senescence. Thus, Pep-triggered acceleration of dark-induced senescence is not a result of PTI activation but we discovered a unique feature of the Pep-PEPR system, further separating the Pep response from the typical MAMP response.

### Pep-triggered acceleration of starvation-induced senescence is mediated by the activation of chlorophyll breakdown and autophagy

Continuous darkness is known to cause energy deprivation and subsequently promotes starvation (Baena-Gonzalez and Sheen, 2008). Plants are able to recover after a certain period of starvation but eventually senesce and die if the situation of starvation persists (Avila-Ospina et al., 2014). A key element of survival during times of starvation is the remobilization of nutrients. Chloroplasts for example are rich nutrient sources since they store starch and contain most of the leaf nitrogen. Therefore, leaf yellowing is often a sign for chloroplast decay and nutrient remobilization (Ren et al., 2014). Nutrient remobilization is mainly implemented by the autophagy machinery (Liu and Bassham, 2012). Any alterations of this fine-tuned system accelerate starvation-induced as well as age-dependent senescence (Hanaoka et al., 2002; Xiao et al., 2010).

**Figure 8:**
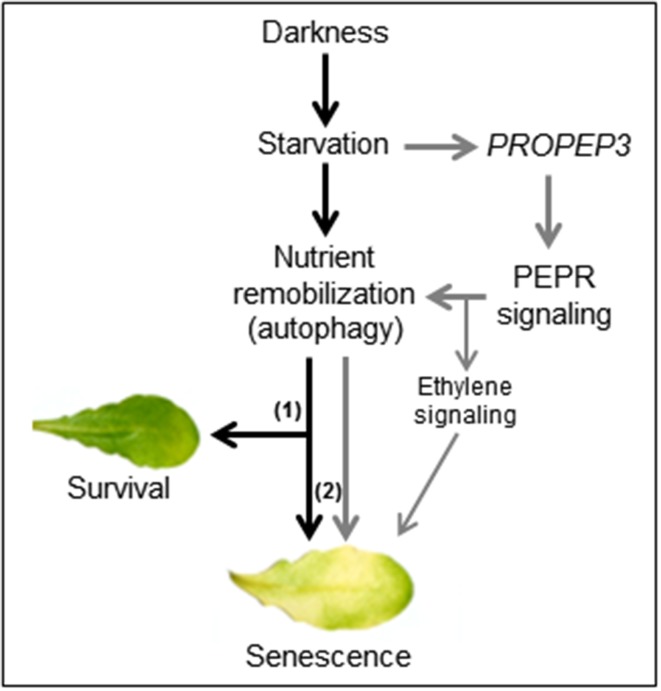
**A working model for the impact of Pep-PEPR-signaling on starvation-induced senescence**. Continuous darkness leads to starvation due to energy deprivation and lack of nutrients. In order to survive nutrients are remobilized e.g., by induction of autophagy. During short periods of starvation this response supports tissue survival (1) whereas continuous starvation and autophagy (2) eventually leads to senescence. In our study we found that darkness/starvation induces *PROPEP3* transcription. We hypothesize that this might also promote the release of mature Pep3, which activates PEPR signaling to fine tune nutrient remobilization of responsive cells via chlorophyll breakdown and autophagy. In case of the leaf discs or detached mature leaves, supplementation of the assay solution with Peps triggers a detrimental turnover of cellular components, thus shortens the time of survival and accelerates the onset of senescence during continuous darkness.

We observed that on the one hand continuous darkness led to a rise in *PROPEP3* transcript levels and on the other hand PEPR signaling induced genes encoding key enzymes for chlorophyll breakdown (*PAO*) as well as autophagy (*APG7* and *APG8a*) (Pruzinska et al., 2003; Ren et al., 2014). The latter three genes are already known to be induced upon prolonged darkness (Buchanan-Wollaston et al., 2005; Avila-Ospina et al., 2014) but here we found a stronger and/or more rapid induction in the simultaneous presence of Peps. Thus, it seems that the Pep-PEPR system is induced upon the advent of starvation and that PEPR signaling might contribute to activate nutrient remobilization. This conclusion seems counterintuitive at first since nutrient remobilization upon starvation stress is meant to enhance survival and delay senescence. But the machinery behind nutrient remobilization is precisely adjusted. Modifications like the knock-out or the overexpression of *APG* genes both result in an early senescence phenotype (Hanaoka et al., 2002; Slavikova et al., 2008; Liu and Bassham, 2012; Li et al., 2014). Based on the observed induction of *PROPEP3* but not *PROPEP1* during continuous darkness we hypothesize that the abundance of individual PROPEPs and the release of their respective Peps might be precisely regulated as well to fine-tune the onset of senescence via activation of autophagy and subsequent nutrient remobilization. In contrast, the external supplementation of Peps leads to a hyperinduction of the autophagy machinery with the already described consequence of an early senescence phenotype (Liu and Bassham, 2012). We summarized this in our simplified working model (Figure 8). However, the contribution of the Pep-PEPR system to the regulation of nutrient remobilization via autophagy in order to prevent an early onset of senescence seems either little or limited to special circumstances since we hitherto could not find a senescence phenotype of the *pepr1 pepr2* double mutant, which is completely impaired in PEPR-mediated signaling. In line with this idea is our observation that young leaves did not show an early senescence phenotype upon Pep-treatment, probably due to the lack of competency to senesce (Thomas, 2013). More research is needed to further unveil the backgrounds of this Pep-PEPR specific effect. With our study we provide first experimental evidence that the Pep-PEPR system has more roles than just being an amplifier of immunity. We believe that our findings will fuel research on the Peps as well as related peptides, and bring new ideas about how plants integrate abiotic stress information and pathogen resistance.

### Conflict of interest statement

The authors declare that the research was conducted in the absence of any commercial or financial relationships that could be construed as a potential conflict of interest.
